# A dataset to assess providers׳ knowledge and attitudes towards the 2013 American College of Cardiology/American Heart Association Cholesterol Management Guideline

**DOI:** 10.1016/j.dib.2016.02.082

**Published:** 2016-03-09

**Authors:** Yashashwi Pokharel, Lynne Steinberg, Winston Chan, Julia M. Akeroyd, Peter H. Jones, Vijay Nambi, Khurram Nasir, Laura Petersen, Christie M. Ballantyne, Salim S. Virani

**Affiliations:** a Sections of Cardiovascular Research, Baylor College of Medicine, Houston, TX, USA; b Department of Medicine, Baylor College of Medicine, Houston, TX, USA; c Center for Cardiovascular Disease Prevention, Methodist DeBakey Heart and Vascular Center, Houston, TX, USA; d Department of Psychology, University of Houston, Houston, TX, USA; e Health Policy, Quality & Informatics Program, Michael E. DeBakey VA Medical Center Health Services Research & Development Center for Innovations, Houston, TX, USA; f Michael E. DeBakey VA Medical Center, Houston, TX, USA; g Center for Prevention and Wellness Research, Baptist Health Medical Group, Miami Beach, FL, USA

**Keywords:** Cholesterol, Guideline

## Abstract

We previously examined provider׳s understanding of the 2013 American College of Cardiology/American Heart Association (ACC/AHA) cholesterol management guideline (DOI: http://dx.doi.org/10.1016/j.jacl.2015.11.002)(Virani et al., 2013) [1], and also assessed whether a case-based educational intervention could improve providers׳ knowledge gaps and attitudes towards the guideline (DOI: 10.1016/j.atherosclerosis.2015.12.044) (Pokharel, et al., 2016) [2]. Here we describe the dataset that we used to examine our objectives.

**Specifications Table**

**Table:** 

Subject area	*Biology, Psychology*
More specific subject area	*Lipidology and behavior*
Type of data	*Multiple choice questions in a questionnaire developed using a conceptual model*[3]
How data was acquired	*Survey, paper-based*
Data format	*Questions tested for validity*
Experimental factors	*Pre-tested including one-on-one session*
Experimental features	*Pre-tested in 11 providers followed by one-on-one session between a psychometrician and a provider for each question*
Data source location	*Houston, Texas, USA*
Data accessibility	*Data within this article*

**Value of the data**

• Our tool (dataset) can be used for data collection by researchers and educators to assess provider׳s understanding towards the 2013 ACC/AHA cholesterol guideline
• The tool is designed to assess provider׳s understanding of the guideline in three domains – knowledge, attitude and behavior towards the 2013 ACC/AHA cholesterol guideline, and it also provides information about the current practice and perceived barriers towards implementation of the 2013 ACC/AHA cholesterol guideline
• The tool can also be used to understand the effectiveness of an educational intervention by comparing change in responses before and after the educational intervention
• The tool can be used for quality improvement initiatives or as part of a research study within one or more institutions for providers who are in training or currently practicing.

## 1 Data

The tool (dataset) consisted of a questionnaire containing providers׳ demographic information followed by the actual survey questions. There were a total of 23 multiple-choice questions and 2 clinical vignettes [1], [2].

## 2 Experimental design, materials and methods

Cabana et al. have described a conceptual model as a framework to identify barriers to implementation of clinical practice guidelines, [3] which have been used in prior studies related to cholesterol guidelines [4], [5]. We identified providers׳ knowledge, attitudes and behaviors as the three domains related to barriers to implementation of the 2013 ACC/AHA cholesterol management guideline as described in Cabana׳s model [3]. The questions related to each domain were developed to identify the salient features of the 2013 ACC/AHA cholesterol guideline [6] and to distinguish these features from the preceding guideline (National Cholesterol Education Program – Adult Treatment Panel III [ATP-III] report) [7], [8]. We developed this tool with the help of a psychometrician and pre-tested among physician-in-training, physician-in-practice and physician assistant providers from internal medicine, cardiology and endocrinology disciplines. Subsequently, a psychometrician conducted a one-on-one session with an internal medicine provider to ensure the fidelity of the questions in the tool. We also included some filler items to ensure that the purpose of the survey was less apparent to the respondents and therefore, their responses would not be affected. Fig. 1 links each question to one of the 3 domains. A filled questionnaire sample data is presented with this article.

In the knowledge domain, we asked whether providers were familiar with the following - the 2013 ACC/AHA guideline; the 4 groups of patients who could potentially benefit from statin therapy (those with clinical atherosclerotic cardiovascular disease [ASCVD], low density lipoprotein cholesterol [LDL-C]≥190 mg/dL, patients with diabetes aged 40–75 years, 10-year ASCVD risk ≥7.5%); the 10-year ASCVD risk estimator; outcomes calculated by the 10-year ASCVD risk estimator and the differences between the 10-year ASCVD risk estimator and the Framingham coronary heart disease risk estimator recommended by the prior ATP-III guideline; ASCVD risk threshold for initiating discussion regarding benefit and risk of statin therapy; providers’ awareness that LDL-C≥190 mg/dL could identify patients with genetic hyperlipidemia, such as familial hypercholesterolemia (FH); definition of statin dose intensity; and providers’ knowledge regarding the need to measure lipids after statin initiation to assess response and adherence to lifestyle and pharmacotherapy.

In the attitude domain, we asked providers׳ agreement with the following: the 2013 ACC/AHA guideline; belief that use of the new 10-year ASCVD risk estimator will either under- or overestimate the true 10-year ASCVD risk in their patients; belief that the provider cannot perform guideline-recommended care; belief that following the 2013 ACC/AHA guideline will not improve cardiovascular outcomes in his/her patients; and whether providers will continue to use a LDL-C target based treatment approach.

In the behavior domain, we assessed organizational/practice barriers (type of provider [specialist or primary care], provider-in-training or provider-in-practice, academic vs. private practice setting, number of years since completion of formal training), and barriers perceived by providers as secondary to patient factors (e.g. inability to afford medications and non-adherence to lipid lowering medication) as potential reasons for their inability to effectively follow the 2013 ACC/AHA cholesterol guideline.

The questionnaire included two clinical vignettes. The first case asked providers for a recommended treatment plan for a patient with LDL-C of 210 mg/dL (likely heterozygous FH). In the second case, providers were asked whether they would measure a lipid panel in a patient with a recent myocardial infarction who was started on statin therapy to assess treatment response and adherence.

We administered the questionnaire to providers at local educational conferences at their own institutions or at regional continuing medical educational activities at 21 sites between September 2014 and April 2015. The response rate was 72.1%; 58% were men, 43% were trainee providers, 49% were in internal medicine and 27% in cardiology specialty, and 54% practiced in academic setting [1].
