# Supplementary material for: A dataset to assess providers׳ knowledge and attitudes towards the 2013 American College of Cardiology/American Heart Association Cholesterol Management Guideline
Source: Data Brief. 2016 Mar 9;7:595–8. doi: 10.1016/j.dib.2016.02.082 (PMC4802420; doi:10.1016/j.dib.2016.02.082)
Supplement: Supplementary file 1 — Supplementary material [file mmc1.docx]

**Funding**: This work was supported by Baylor College of Medicine Academy of Distinguished Educators Fulbright & Jaworski L.L.P. Educational Grant. Dr. Pokharel is supported by the National Heart, Lung, and Blood Institute of the National Institutes of Health under Award Number T32HL110837. Dr. Virani is supported by the American Heart Association Beginning Grant-in-Aid (14BGIA20460366) and the American Diabetes Association Clinical Science and Epidemiology award (1-14-CE-44). This work was also supported by the Houston VA HSR&D Center for Innovations grant (grant HFP 90-020).

**Role of the Funder/Sponsor**: The funding sources had no role in study design; in the collection, analysis, and interpretation of data; in writing the report; and in the decision to submit the article for publication.
