# Supplementary material for: A dataset to assess providers׳ knowledge and attitudes towards the 2013 American College of Cardiology/American Heart Association Cholesterol Management Guideline
Source: Data Brief. 2016 Mar 9;7:595–8. doi: 10.1016/j.dib.2016.02.082 (PMC4802420; doi:10.1016/j.dib.2016.02.082)
Supplement: Supplementary file 2 — Supplementary material [file mmc2.zip › Dataset.pdf]

Clinical Practices for Cholesterol Management Questionnaire

We would like to know your views on the recently released American College of Cardiology (ACC)/ American Heart Association (AHA) Guidelines on the Treatment of Blood Cholesterol to Reduce Atherosclerotic Cardiovascular Risk in Adults. We greatly appreciate your taking 5-10 minutes to complete this questionnaire.

Age: 38 yrs ***Gender***: ☒ Male ☐ Female

Your current position: ☒ Physician ☐ Nurse Practitioner ☐ Physician Assistant ☐ Physician in training ☐ Other (Please specify)_________

Specialty: ☒ Internal Medicine ☐ Family Practice ☐ Endocrinology ☐ Cardiology ☐ Other (please specify)_________

<u>If you are in training</u>: Year of residency if you are a resident: 3
Year of fellowship if you are a fellow: _________
Type of residency or fellowship program: ☐ Private ☒ Academic ☐ Private with an academic affiliation

<u>If you are in practice</u>: Number of years since completing residency or fellowship: ______
Practice area: ☐ Outpatient only ☐ Inpatient only ☐ Both inpatient and outpatient
Type of practice: ☐ Private ☐ Academic ☐ Private with an academic affiliation

1. Which of the following best describes your knowledge of the 2013 ACC/AHA cholesterol management guideline?
   - ☐ a. I am not aware of this guideline.
   - ☐ b. I am aware of its existence, but not aware of its contents.
   - ☒ c. I am aware of some of the content but have not read the summary or the full report.
   - ☐ d. I have read the summary.
   - ☐ e. I have read the full report.

Now, we would like to ask you questions regarding the 10-year atherosclerotic cardiovascular disease (ASCVD) Pooled Cohort Equation recommended by the recent cholesterol management guideline:

2. How often do you use the ASCVD 10-year risk calculator in your practice?
☐ a. Routinely ☒ b. Sometimes ☐ c. Rarely ☐ d. I do not use it

3. Are you aware of the web version or the downloadable ASCVD 10-year risk calculator?
☒ a. Yes ☐ b. No
If yes, then how do you access it: ☐ Desktop ☐ Laptop ☒ Smartphone ☐ iPad ☐ Electronic health record

4. *<u>If answer to question 3 is yes</u>*, how comfortable are you in using the ASCVD 10-year risk calculator?
☐ a. Very comfortable ☒ b. Somewhat comfortable ☐ c. Not comfortable

5. Positive family history of premature cardiovascular disease is a factor used in the new ASCVD risk calculator
☐ a. Yes ☒ b. No

6. The current guideline recommends discussing the use of a statin with a patient at what 10-year ASCVD risk threshold?
☐ ≥4% ☒ ≥7.5% ☐ ≥10% ☐ ≥15% ☐ ≥20%

7. The 10-year ASCVD risk calculator provides an estimated risk of what <u>outcome(s)</u> (*please check all that apply*)?
☒ Fatal myocardial infarction (MI) ☒ Non-fatal MI ☐ Need for stenting or bypass surgery ☒ Fatal stroke
☒ Non-fatal stroke ☐ Death from any cause

8. Are you aware of any differences between the new 10-year ASCVD risk calculator and the prior Adult Treatment Panel (ATP) III Framingham risk calculator (*please check all that apply*)?

☒ a. The new calculator is race specific, whereas, the prior risk calculator was not.

☐ b. The new risk calculator includes family history in calculation of 10-year risk, whereas the prior calculator did not.

☒ c. The 10-year adverse outcome(s) estimated by the new risk calculator differ from the prior calculator.

☐ d. They are the same except that the threshold for treatment consideration is lower for the new 10-year ASCVD risk calculator compared with the prior risk calculator.

Instructions: Please indicate how strongly you agree with each of the following statements by circling the appropriate number on the response scale.

	**Strongly Disagree**	**Somewhat Disagree**	**Neutral**	**Somewhat Agree**	**Strongly Agree**
9. I have confidence in the new ACC/AHA cholesterol guidelines.	1	2	3	4	(5)
10. I have confidence in the new ACC/AHA ASCVD risk calculator.	1	2	3	4	(5)
11. The use of the ACC/AHA 10-year ASCVD risk calculator will overestimate the true 10-year CVD risk in my patients.	1	(2)	3	4	5
12. The use of the ACC/AHA 10-year ASCVD risk calculator will underestimate the true 10-year CVD risk in my patients.	(1)	2	3	4	5
13. Following the new ACC/AHA cholesterol guideline will lead to an improvement in cardiovascular outcomes in my patients.	1	2	3	(4)	5
14. With recent guidelines, I do not see a need to repeat a lipid panel once a patient is on a statin.	(1)	2	3	4	5

15. I usually titrate the statin dose on my patients rather than starting them on the highest recommended dose.

☒ a. Yes ☐ b. No

16. Do you currently use LDL cholesterol as the *target of therapy* for cholesterol management in your patients?

☒ a. Yes ☐ b. No

17. Approximately, what percent of your patients are not able to tolerate statins due to myalgias? 20%.

18. A 30 year old White male with no prior medical history presents to your office for a routine physical exam. His lipid panel shows a total cholesterol of 265 mg/dL, triglycerides of 80 mg/dL, LDL-C of 210 mg/dL, and HDL-C of 39 mg/dL. What would you recommend?

☐ a. Screening with carotid intima media thickness or coronary calcium score.

☒ b. Discuss the use of statin therapy with the patient.

☐ c. Perform a 10-year ASCVD risk calculation to determine if patient is a candidate for statin therapy.

☐ d. Discuss diet and life style recommendations and repeat 10-year ASCVD risk calculation in 5 years.

☐ e. Reassure the patient and repeat lipids and 10-year ASCVD risk calculation in 5 years.

19. A 63 year old African American male with history of hypertension (treated with hydrochlorothiazide) presents for a routine check-up. His fasting lipid panel shows total cholesterol 183 mg/dL, triglycerides of 85 mg/dL, HDL-C of 42 mg/dL, LDL-C 124 mg//dL. He is not a smoker or a diabetic. His blood pressure is 127/84 mm Hg. What is your guess about this person's 10-year risk of ASCVD?

☐ a. <5% ☐ b. 5-7.5% ☐ c. 10% ☒ d. 15% ☐ e. 20% ☐ f. >20%

20.The recent cholesterol management guideline recommends discussing statin use with which group of patients?

☐ a. Patient with established cardiovascular disease, diabetics age 40-75 years, patients with chronic kidney disease on hemodialysis, and patient with LDL cholesterol ≥ 190mg/dL

☐ b. Patients with established cardiovascular disease, diabetics age 40-75 years, patients with LDL cholesterol ≥ 190mg/dL, and patients with 10 years ASCVD risk ≥ 10%

☐ c. Patients with established cardiovascular disease, diabetics age 40-75 years, patients with LDL cholesterol ≥ 190mg/dL, and patients with chronic kidney disease not on dialysis

☒ d. Patients with established cardiovascular disease, diabetics age 40-75 years, patients with LDL cholesterol ≥ 190mg/dL, and patients with 10 years ASCVD risk ≥ 7.5%

21. What percent of LDL-C reduction would you expect from low, moderate or high intensity statin therapy according to the 2013 ACC/AHA cholesterol management guideline?

☐ (a) <20% for low, 20-<40% for moderate, and ≥ 40% for high-intensity statin therapy.

☐ (b) <30% for low, 30-<40% for moderate, and ≥ 40% for high-intensity statin therapy.

☐ (c) <20% for low, 20-<35% for moderate, and ≥ 35% for high-intensity statin therapy.

☒ (d) <30% for low, 30-<50% for moderate, and ≥ 50% for high-intensity statin therapy.

☐ (e) I do not know.

22. You are evaluating a 55 year old African American patient 4 weeks after admission for a ST segment elevation myocardial infarction. Patient received a stent. The patient was also started on aspirin 81 mg daily, clopidogrel 75 mg daily, metoprolol 25 mg twice daily, and atorvastatin 80 mg daily. At this point:

☒ (a) You will repeat a fasting lipid panel in the next 6-8 weeks.

☐ (b) You will repeat a fasting lipid panel in the next 12-15 months.

☐ (c) You will not repeat a lipid panel since the patient is already on high-intensity statin therapy.

23. What are the possible reasons that you may not be able to follow the cholesterol management guideline in your practice? Below we have listed many possible reasons. *Please check all that apply.*

☐ (a) I am not very familiar with the recent cholesterol management guideline.

☐ (b) I do not agree with the treatment recommendations made by the recent cholesterol guideline.

☐ (c) I am not aware of the data supporting the recent cholesterol management guidelines.

☐ (d) I am aware of the data but do not believe that cholesterol guideline is important to follow in my patients.

☐ (e) I do not agree with the new 10-year ASCVD risk calculator.

☒ (f) I have heard in the media that the use of new 10-year ASCVD risk calculator will lead to statin overuse.

☒ (g) I do not have time to follow guidelines in my practice.

☐ (h) I do not know where to look for the recent guidelines.

☐ (i) I do not know where to look for the recent 10-year ASCVD risk calculator.

☒ (j) My patients will not be compliant with statins due to side effects (e.g. myalgia).

☐ (k) The medication cost associated with statins in my patients will be prohibitive.

☐ (l) Other reasons (*please specify*): ____________

Any comments or concerns about the questionnaire or the guideline? Please comment on the reverse side.

Thank you for participating in our study!
